# Research Priorities and Resource Allocation in the Investigation of New Drugs for Cancer in Least Developed Countries

**DOI:** 10.1155/2018/8092702

**Published:** 2018-07-02

**Authors:** Ricardo Eccard da Silva, Angélica Amorim Amato, Débora Dornelas Belchior Costa Andrade, Alessandra Vanessa Leite e Silva, Marta Rodrigues de Carvalho, Maria Rita Carvalho Garbi Novaes

**Affiliations:** ^1^Office of Clinical Trials, Brazilian Health Regulatory Agency (ANVISA), Setor de Indústria Trecho 5, Área Especial 57, 71205-050, Brasília, Brazil; ^2^Faculty of Health Sciences, University of Brasilia (UnB), Campus Universitário Darcy Ribeiro, 70910-900 Brasília, Brazil; ^3^Secretary of Health, Government of the Federal District, Quadra 45, Vila São José (Brazlândia), 72745000 Brasília, Brazil; ^4^Hospital de Base, Secretary of Health, Government of the Federal District, SMHS, Quadra 101, Área Especial, s/n, Asa Sul, 70330-150, Brasília, Brazil; ^5^School of Medicine, Health Science Education and Research Foundation (FEPECS), SMHN Quadra 03, Conjunto A, Bloco 1, Edifício FEPECS, 70.710-907 Brasília, Brazil

## Abstract

Cancer incidence has increased significantly in low- and middle-income countries. The priorities of international health research are not always aligned with the global burden of cancer. This study aims to analyze global tendencies in clinical trials in oncology and discuss research priorities and resource allocation in the investigation of new drugs for cancers that significantly affect the least developed countries. This was a retrospective and analytical study that included data collected from the World Health Organization's International Clinical Trials Registry Platform (ICTRP) in 2014. According to our results, there was a tendency for clinical trials involving breast and lung cancer to be conducted in countries with a lower level of economic development. On the other hand, cervical, stomach, and liver cancer, despite the significant burden that these place on middle- and low-income countries, were studied little among the countries selected. In conclusion, the organizations that most fund research to develop new drugs for cancer treatment continue to show little interest in prioritizing resources to fund research for certain types of cancer such as those of the cervix, stomach, and liver, which have a significant impact in low- and middle-income countries.

## 1. Introduction

In high-income countries (HIC), as defined by gross national income per capita, the incidence of cancer continues to be high, particularly for lung, breast, colorectal area, and prostate. Although the mortality rate has reduced in these countries, it has increased in low- and middle-income countries (LMIC). In these countries, an increase in the incidence of cancer of the stomach, liver, esophagus, and cervix has been observed. Although certain types of cancer, such as cervical cancer, disproportionately affect less-developed regions, such as Latin America, they have been studied little in these regions [1, 2].

In the LMIC, the epidemiological transition influences the changes in the patterns of mortality and causes of death, characterized by the transition from infectious diseases to the noncommunicable diseases, such as cardiovascular disease, diabetes, and cancer. Due to greater exposure to risk factors such as smoking, sedentarism, obesity, and the aging of the population in LMIC, the proportion of new cases of cancer diagnosed is expected to increase from 59% in 2012 to 65% by 2030 [3].

In 2015, 8.8 million people died from cancer, which was the second greatest cause of death worldwide. Approximately 70% of these deaths occurred in LMIC [4]. Worldwide, there were 2 million cases of cancer of the bronchi and lungs and 1.7 million people died due to this form of cancer in 2015. In that same year, the incidence and mortality for other types of cancer, respectively, were breast (2.4 million; 533,000 people), colorectal area (1.7 million; 832,000 people), prostate (1.6 million; 366,000 people), stomach (1.3 million; 819,000 people), liver (854,000; 810,000 people), and cervix (526,000; 239,000 people) [5].

In relation to the global cancer burden, the difference between HIC and LMIC is found mainly in two aspects: firstly, the high global burden of cancer in LMIC is related to forms of cancer related to infection, such as that of the stomach, liver, and cervix. Secondly, due to the epidemiological transition, cancers of the lungs, colon, and breast are increasing rapidly in LMIC. On the other hand, reductions have been observed in the HIC in the rates of incidence and mortality from cancers of the lungs, breast, cervix, and prostate [3].

Cancers of the stomach, liver, and cervix have an important impact on mortality in LMIC in comparison with HIC. The number of deaths among individuals aged from 0 to 69 years in both sexes in countries classified by level of economic development in 2012 may be separated as follows: stomach (HIC: 60,000; LMIC: 300,000), liver (HIC: 50,000; LMIC: 400,000), and cervix (HIC: 20,000; LMIC: 200,000) [6].

In LMIC, 26% of all types of cancer are related to infectious agents, such as human papilloma virus [7]. The differences between HIC and LMIC in relation to the incidence of specific types of cancer, such as cancer of the cervix, are related to the implementation and extension of efficient screening programs, such as the smear test [8].

Difficulties related to prevention and control of risk factors and to screening programs for detecting cancers in the early stages are present in the LMIC. The number of cases and mortality due to cancer of the stomach, liver, and cervix could be reduced with programs for prevention and early detection extended in these countries. It is also necessary to improve the treatment infrastructure and access to palliative care in these countries [7].

World health research is determined more by economic and marketing interests than by health priorities or disease burden [9]. Around 90% of the resources spent annually on medical and health-related research focus on the health needs of the richest 10% of the world's population, while only the remaining 10% of the resources are directed towards the needs of the remaining 90% of the population [10]. Unsurprisingly, given that the vast majority of studies are financed by the richest countries, most studies investigate rich countries' health needs. Worldwide, research is aligned with the global market for treating diseases which are related to financial return. In contrast, conditions which are unlikely to generate revenue, but which disproportionately impact the health of populations in LMIC, are less likely to attract the attention of organizations financing studies [11].

The neglected diseases, which mainly affect low-income countries, have been of little interest to those financing research for developing new drugs. One already published study showed that among the therapeutic products approved in 2000–2011 in various countries only 4% were for these conditions, although the global health burden resulting from these diseases is estimated at 11% [12].

In regions with limited resources, such as Africa, the number of new cases of cancer has increased each year. This number is expected to increase by 60% by 2030. Although being a public health problem in this region, cancer is not yet a priority in health care programs or in research into new forms of treatment. In African countries, cancers of the liver, cervix, and bladder, along with Kaposi Sarcoma, are among the most prevalent ones [13, 14]. Clinical trials financers, however, have directed few resources towards conducting studies on these types of cancer.

The 2030 Agenda of the World Health Organization (WHO) for Sustainable Development considers the control of these conditions to be a major challenge and recommends that countries prioritize investment in the prevention and reduction of deaths [15].

The present study aimed to analyze the global tendencies of clinical trials in oncology and to discuss research priorities and allocation of resources in the investigation of new drugs for cancers that significantly affect the least developed countries.

## 2. Methods

### 2.1. Design

The study reported here was a cross-sectional overview. The data was obtained from the International Clinical Trials Registry Platform (ICTRP) database. The ICTRP accepts data from a total of 16 national and regional registries from various countries that meet their quality criteria, including the ClinicalTrials.gov of the United States and the EU Clinical Trials Register of the European Union [16]. It considers studies registered in the ICTRP between 01/01/2014 and 12/31/2014. The period of data collection was 01/03/2014 to 06/31/2015. Due to the limited number of studies in African countries over the one-year period, for these countries the selected period was 01/01/2006 to 12/31/2016. The period of data collection was the same.

### 2.2. Selection Criteria

#### 2.2.1. Inclusion Criteria

These include clinical trials in oncology, registered in the ICTRP, that involved drug interventions in countries with the highest average rates of growth in numbers of trials, highest trial densities, and greatest trial capabilities.

#### 2.2.2. Exclusion Criteria

These include observational studies and studies on medical devices and procedures.

### 2.3. Selected Variables

These include health condition, according to the International Classification of Diseases (ICD-10); the study population by age range; the study's sponsor; and phase of development.

Clinical trials were selected from countries with the

- highest trial densities during 2005 to 2012, based on the trial density (annual number of registered clinical trials divided by country population in 2010), Denmark (106.9), Estonia (86.8), Netherlands (73.7), Israel (67.5), and Finland (63.1) [18], and trial density (the number of recruiting sites on April 12, 2007, divided by the country population in millions), the United States (120.3), Germany (51.2), France (50.3), Spain (46.4), and Italy (34.6) [19],
- highest average growth rates in clinical trials (2005 to 2012), Iran (196.6%), China (43.5%), Egypt (35.3%), South Korea (34.5%), Japan (33.3%), India (32.4%), Brazil (19.3%), Turkey (18.6%), Ukraine (16.9%), Colombia (15.4%), Singapore (11.6%), Russia (10.7%), Thailand (9.9%), and Malaysia (9.5%) [18], and highest average growth rate in number of trial sites, based on the number of clinical sites completed or actively recruiting on April 12, 2007, Peru (32.5), the Philippines (30.9), Argentina (26.9), Mexico (22.1), and Chile (10.6) [19],
- greatest trial capabilities (calculated as the average number of clinical sites per trial that each country contributed in large trials), Japan (33.4), United Kingdom (7.6), and South Africa (4.3) [19].

The African countries (Zimbabwe, Malawi, Mozambique, Algeria, Mauritius, Mali, Tunisia, Libya, Uganda, Congo, Kenya, and Nigeria), where cancer has a high incidence and significant impact on the health of the population, were also selected [13].

**Figure 1:**
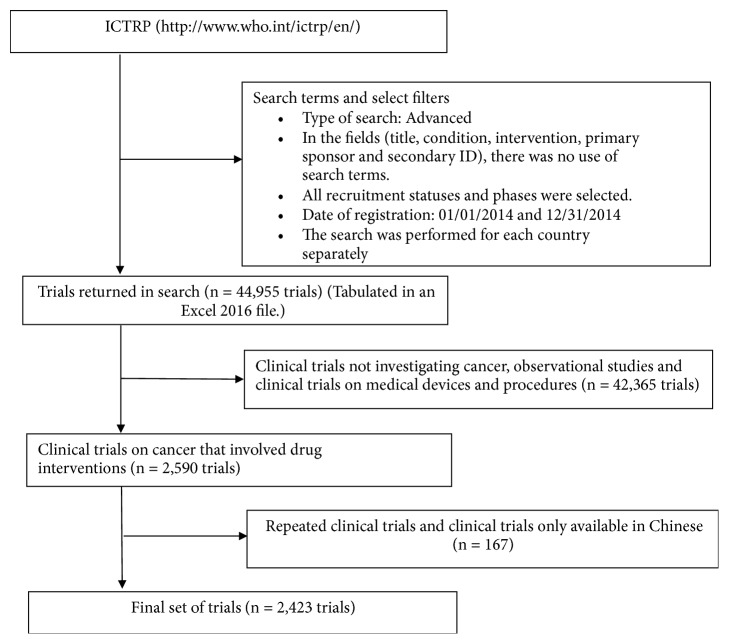
Study selection criteria, adapted figure [17].

Figure 1 contains the steps for searching studies on the platform.

No bias control procedure was used.

There were studies that investigated several cancer types, and the clinical condition was stated as “neoplasm”. In these situations, clinical condition was classified as “C00-C97”.

### 2.4. Statistical Analysis

We hypothesize that cancers which have the greatest impact on health outcomes in LMICs have not been well studied in global multicenter clinical trials. The Kruskal-Wallis test [20–22] was used to analyze these tendencies and associations with different levels of development. Later, the Jonckheere [20] test was used. The statistical software used was the R Statistical Software [21].

### 2.5. Analysis

Age range was classified according to the United States' National Institute of Health (NIH): 80+ years = 80 and over; 65+ years = aged; 45-64 years = middle-aged; 19-44 years = adult; 13-18 years = adolescent; 6-12 years = child; 2-5 years = preschool child; 1-23 months = infant; birth-1 month = newborn [23].

The countries were classified according to the World Bank's classification of their economic level [24] by 2016 Gross National Income (GNI) as follows:

- Low-income countries: GNI per capita of US$1,025 or less
- Lower middle-income countries: GNI per capita between US$1,026 and US$4,035
- Upper middle-income countries: GNI per capita between US$4,036 and US$12,475
- High-income countries: GNI per capita above US$12,476.

The study sponsor was classified according to the information on the organization's website. The primary sponsor is defined in the WHO ICTRP as the “organization which takes responsibility for the initiation, management, and/or financing of a clinical trial” [25].

The study was approved by the Research Ethics Committee of the Health Sciences College of the University of Brasília (Brazil).

## 3. Results

The search in the ICTRP returned 44,955 studies. After this, only cancer drugs clinical trials were selected (n=2,590). 145 studies from China were excluded, because the information was only available in Chinese. A further twenty-two studies were excluded because they were duplicated; that is, they were registered more than once in the same country. After eliminating these studies, 2,423 studies were evaluated.

### 3.1. Trends of Clinical Trials on Cancer in Countries Classified by Level of Economic Development

There was a trend for the percentage of studies involving breast and lung cancer to reduce as the level of development increases, at a level of significance of 5% (p<0.05). Although the malignant neoplasm of the rectosigmoid junction had a value of p<0.1, it was not possible to conclude that there was a trend in relation to the countries' level of economic development, as the distribution of the data of the HIC showed high variability. The cancer types (bladder, liver cell carcinoma, cervical, and brain) had few observations and the Kruskal-Wallis test could not be performed.

Malignant neoplasms of ovary and head and neck had a low p value (but not less than 5%). The problem was, however, that the small amount of data representing the group of LMIC made it impossible to use the Kruskal-Wallis statistical test.

**Figure 2:**
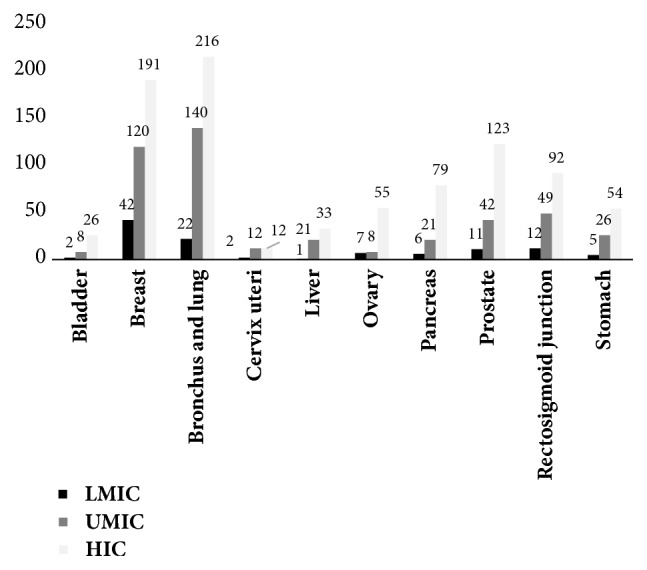
Number of clinical trials by type of cancer and countries' incomes.

Figure 2 shows that lung and breast cancer were the most studied in all of the countries selected, regardless of level of economic development. The least-studied types of cancer were liver, bladder, and cervical cancer.

### 3.2. Financing of Cancer Trials

**Figure 3:**
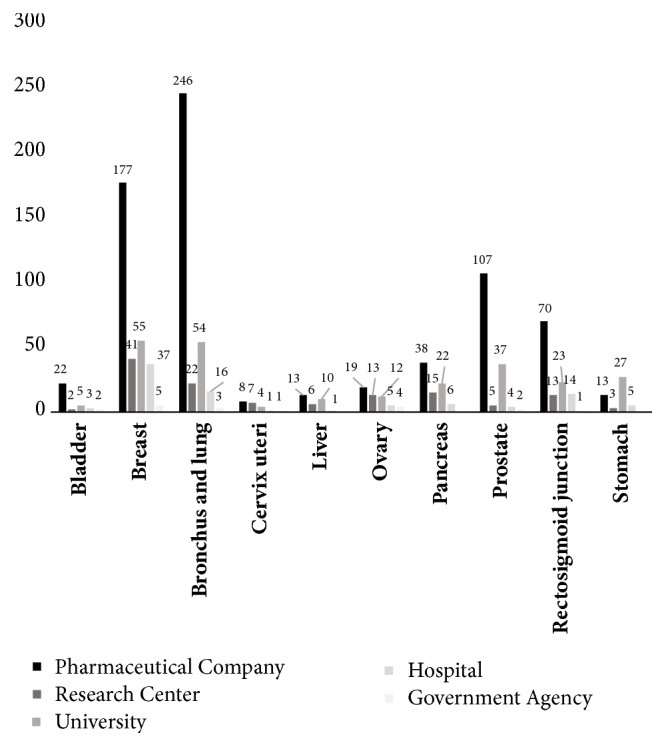
Number of clinical trials by type of cancer and study sponsor.

Figure 3 shows that the cancers of the breast, lungs, prostate, and rectosigmoid junction were most studied by pharmaceutical companies. On the other hand, stomach cancer was most studied by universities. Government agencies sponsored few studies.

### 3.3. Cancer Clinical Trials in Africa

In a ten-year period, few studies to develop new drugs for cancer treatment were conducted in African countries. From 2006 to 2016 there were 12 studies in Algeria (breast, thyroid, large B-cell lymphoma, renal, gastric, head and neck, nasal, and rectosigmoid junction), 1 study in Libya (liver), 2 studies in Malawi (Burkitt's lymphoma), 3 studies in Nigeria (breast), 23 studies in Tunisia (breast, lungs, nasal, gastric, prostate, renal, head and neck, pancreatic, and rectosigmoid junction), and 1 study in Zimbabwe (cervical). There were no studies in Congo, Kenya, Mali, Mauritius, Mozambique, or Uganda.

### 3.4. Phase of Development

Phase I clinical trials are concentrated more heavily in regions such as the United States, Japan, and Europe (Table 1).

### 3.5. The Pediatric Population in Clinical Trials

**Figure 4:**
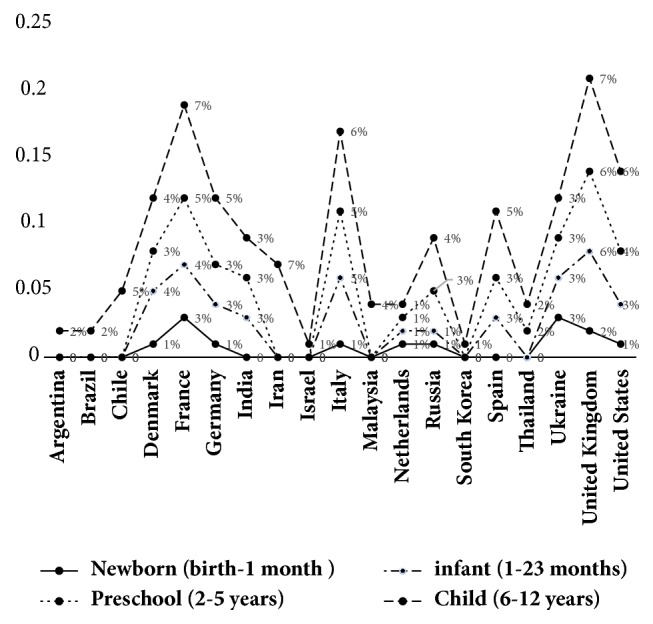
Percentage of clinical trials by type of pediatric age group.

Figure 4 shows that studies involving the pediatric population were concentrated more heavily in Denmark, France, Germany, Italy, Russia, Spain, Ukraine, the United Kingdom, and the United States. By far the majority of these countries are of high income. There were no pediatric studies in China, Colombia, Egypt, Estonia, Finland, Japan, the Philippines, Mexico, Peru, Singapore, South Africa, or Turkey.

## 4. Discussion

### 4.1. Addressing of LMIC Health Needs in International Cancer Clinical Trials

The populations of LMIC are increasingly exposed to risk factors such as smoking, obesity, the adoption of sedentary lifestyles, and consumption of industrialized foods, as has already been occurring for some time in HIC [2]. The tendency for research into new drugs for treating breast and lung cancer in countries with a lower level of economic development may be related to greater exposure to risk factors.

According to the World Health Organization, the types of cancer which killed most people in 2015 were lung (1.69 million), liver (788,000), colorectal area (774,000), stomach (754,000), and breast (571,000) [4]. In relation to the incidence of cancer per 100,000 individuals in LMIC selected in the study, breast cancer was the type with the largest number of cases in 2012 in Egypt (49.5), India (25.8), the Philippines (47.0), and Ukraine (41.3). In the same year, there were another types of cancer among those with the highest incidence: Egypt (liver, 25.6, and bladder, 13.1), India (cervical, 22.0, and oral cavity, 7.2), the Philippines (lungs, 19.3, and prostate, 18.0), and Ukraine (colorectal area, 23.4, and lungs, 22.2) [26].

According to our study's results, the types of cancer studied most in clinical trials were lungs, breast, prostate, and colorectal area, at all levels of income. If one considers the types of cancer which cause the most deaths worldwide, such as breast, lung, and colorectal area, the development of new drugs is aligned with these health needs. Besides this, in relation to the types of cancer most common in LMIC, the study of cancer of the breast, lungs, prostate, and colorectal area addresses the health priorities of LMIC.

It is necessary to prioritize investment, such that new drugs for specified types of cancer, such as cervical, liver, and stomach cancer, may be developed. Although breast and lung cancer have important impacts on global health, types of cancers related to less favorable socioeconomic conditions, such as stomach cancer, cervical cancer, and liver cancer despite their increase in developing countries, have been studied little in global medical trials [1, 2].

Although it is the fourth-highest cause of death from cancer worldwide [4], stomach cancer has been studied little in global clinical trials. A large proportion of these deaths is concentrated in LMIC, where the resources for triage are limited [27]. The number of disability-adjusted life years for 2013 was 17.9 million for stomach cancer, below only lung cancer (34.7 million) and liver cancer (20.9 million). For stomach cancer, 77% of this number took place in developing countries [28]. The results of our study showed that only five clinical trials involving this type of cancer were conducted in LMIC.

The present study showed that cervical cancer was studied least. Although reductions in mortality rates from cervical cancer have been achieved in Mexico, this type of cancer continues to cause many deaths in Latin America, approximately 28,000 deaths in this region in 2012. In that same year, there were approximately 36,000 deaths from cervical cancer in the Americas as a whole, 80% of which occurred in Latin America and the Caribbean [29, 30]. One of the countries affected most by this condition is India, where 11.1 per 100,000 deaths are caused by it each year, corresponding to more than 20% of deaths worldwide from this type of cancer [8].

Globally, liver cancer was the fifth most prevalent cancer in 2012 [31] and the second leading cause of cancer death in the world. The patients have short survival rates [4]. This cancer is concentrated in less-developed regions, where about 83% of the cases are diagnosed [24]. This type of cancer is related to 20.9 million disability-adjusted life years; 86% of this is found in developing countries [28].

### 4.2. Financing Clinical Trials

The resources directed towards the prevention, early diagnosis, and treatment of cancer do not take into account this condition's impact in certain regions, as 80% of the disability-adjusted life years lost due to cancer worldwide are found in LMIC, while only 5% or less of global resources are spent in these countries. These regions' populations are increasingly exposed to risk factors such as smoking. If incidence and mortality are to be reduced, it is fundamental to allocate resources in cancer prevention based on controlling these factors. Furthermore, the local governments in these countries spend very little on preventing cancer or on diagnosing and treating it at an early stage, although among the main causes of death worldwide this condition is not included among the global health priorities of the United Nations Millennium Development Goals [32].

The limited resources for financing cancer research must take into account the social burden that each type of cancer involves. If one considers the parameter of “years of life lost by incidence” (calculated based on the division of the number of years of life lost and the number of new cases) cancers of the mouth, uterus, and stomach receive few resources for research in comparison with cancers of the breast and prostate or leukemia. It is the cancers of the bladder, esophagus, mouth, pancreas, liver, stomach, and uterus that receive the fewest public resources for research. These types of cancer, however, have an important impact on the patients' social commitment [14]. In addition, one study showed that there is a disconnection between the mortality rate and the number of clinical trials by cancer site [33]. These types of cancer mainly affect the populations of LMIC. Cancer of the stomach receives the lowest funding for research, equivalent to less than 10% of the sum of the total resources allotted to breast cancer [14].

Our results showed that governmental agencies sponsored few studies. However, data from the United States' National Cancer Institute show that major public investment has been directed towards cancer research. In 2016, $5.38 billion was made available for the National Cancer Institute for research into cancer. The budget spent by the National Cancer Institute for type of cancer in 2015 was for breast (543.7), lung (256.2), prostate (228.9), colorectal area (209.3), pancreas (125.3), ovaries (92.8), liver (70.3), cervix (63.4), and stomach (13.5). Few resources, therefore, have yet been allocated to finance research into types of cancer such as those of the liver, cervix, and stomach, considering the burden caused by these types of cancer in LMIC [34, 35].

Cancer Research United Kingdom has conducted studies on improving treatments and increasing survival for patients with rare types of cancer, such as uveal melanoma and neuroendocrine tumors, as well as cancers of the bladder, cervix, lungs, and esophagus. This last, which is difficult to treat, has been a priority in terms of resource allocation for research [36]. In spite of this, one study indicates that, even in countries such as the United States and the United Kingdom, the amount invested in financing research into specified forms of cancer, such as cancer of the bladder, liver, stomach, lungs, and pancreas, is not proportional to either the social burden or the years of life lost due to these types of cancer in these countries [37].

A separate study that investigated the financing of research in cancer in 2000–2013 by public and philanthropic institutions in the United Kingdom, including the Medical Research Council, showed that there were discrepancies between the social burden of certain types of cancer and the amount invested in research. Cancers of the liver, stomach, esophagus, thyroid, lungs, and bladder, which have substantial social burdens, were underfinanced. The absence of clear criteria for allocating resources may contribute to the inequalities in research and development [38].

In spite of the efforts and investments made by pharmaceutical companies in identifying molecular targets and in research into new drugs [39], the clinical trials for investigating treatments for stomach cancer have been financed by universities, according to the results of our study. Research in universities is fundamental for generating knowledge and for developing innovative products. Such studies tend to be aligned with health needs, promoting the development of medications for neglected or rare diseases. Moreover, universities may implement the Global Access Licensing (GAL) adhering to principles which facilitate access to their innovation for populations with fewer resources [40, 41]. However, types of cancer considered relatively rare, such as cancer of the mouth, continue to be studied little by institutions, including universities [14, 40].

Pharmaceutical companies concentrate their research on new drugs for specific cancers, such as breast or lung cancer or melanoma [42]. One study has shown that private companies tend to invest less in research into conditions with longer survival times, as this leads to delays in commercialization [43]. The greater the survival time is, the longer the study will last, as it is necessary to assess the effects of the experimental drug on improvement in survival. Private companies understand that long studies tend to delay the process of commercializing new drugs [43]. The present study showed that pharmaceutical companies mainly sponsor clinical trials involving cancer of the breast, lungs, prostate, and colorectal area. The data from our study, therefore, contradict those of the already published study mentioned above, as the 5-year relative survival rate for cancer of the breast, prostate, and colorectal area in stage III is approximately 72%, 99%, and 89%, respectively. The rates for cancer of the stomach and liver, on the other hand, in stage IIIA, are 20% and 31% [44].

Financers of clinical trials have shown little interest in undertaking studies for treating cancer in African countries, according to the results of our study. It is important to attract research into cancer in these countries if strategies and treatments related to local needs are to be developed. Due to these countries' limited human and material resources, however, conducting quality studies could be a challenge. Accordingly, it is necessary to encourage programs for developing local skills, through the training of the study team and the development of appropriate research infrastructure. Partnerships with other countries are important in the development of best practices in research [45]. Conducting clinical trials in LMIC may bring benefits besides those related to the new treatment which was tested; such trials may also improve the quality of the medical services and care provided to patients in an institution, regardless of its participation in the study, as the clinical trial may assist in changing the institution's organizational culture through the implementation of systems for ensuring the clinical trial's quality [46]. Although cancers associated with HIV are prevalent in African countries, few resources have been directed towards research into these types of cancers.

On the other hand, participation in clinical trials also involves concerns. One of these is that communities from these regions that participate in clinical trials may not have access to the benefits generated by these studies. One study indicated that out of thirty-three drugs approved for marketing by the US Food and Drug Administration after having been studied in Latin America only eight had been registered and commercialized in all the Latin American countries where they had been tested, and ten had been neither registered nor commercialized in any of the countries. Moreover, drugs studied in Latin America are often not available to most of the population and have few advantages over previously existing interventions [47].

The migration of clinical trials from developed regions to less-developed ones mainly involves phase III studies, which are longer and more expensive and require a large number of study participants. In LMIC, there is greater availability of patients, and the costs involved in the study are lower. The safety profile of new drugs is little-known in the initial phases of clinical development. As a result, the research centers which conduct phase I studies must have appropriate infrastructure, with equipment and research teams that have been trained to provide rapid, quality attendance to the participants, should unexpected events occur involving the use of the drug. The initial phases of clinical trials tend to be concentrated in developed regions, such as the United States, Japan, or Europe, where the research centers generally have better research infrastructure [48]. Our study's results are consistent with this information. Besides the United States, Japan, and European countries, however, our results also showed that Asian countries, such as China and South Korea, have participated more actively in phase I trials than other regions.

In countries with limited resources, as well as shortcomings in research infrastructure, there is also a concern with ethical issues, such as the conducting of phase 1 studies which involve healthy volunteers who could be socially and economically vulnerable [49, 50]. For example, the Latin American region still has 92 million people living in a situation of extreme poverty. Economic vulnerability is related to people with a daily income of between $4 and $10. The incidence of vulnerable people in 2013 was in Brazil (38.4%), Colombia (36.7%), Argentina (34.4%), Chile (37.7%), Mexico (37.8%), and Peru (40.5%) [51]. In addition, there is evidence that unethical practices occur in clinical trials conducted in Egypt by transnational pharmaceutical companies. This country has been the most popular place for testing drugs in the Middle East. Historically, a significant proportion of people living in Egypt have lived in poverty, with only half covered by the public health system. Due to the difficulties in accessing the health system, some people readily accept to participate in a trial as it may be the only way for them to access health care. These factors potentially expose vulnerable people to exploitation as trial participants [52].

Although the incidence of cancer in the pediatric age range is low [53], research into new drugs is necessary in order to improve these patients' clinical condition [54]. Few studies involving the pediatric population have been undertaken among the countries evaluated. According to our study's results, clinical trials involving the pediatric population are more heavily concentrated in HIC, such as the United States, United Kingdom, France, and Italy. These results are consistent with data from a report of the European Medicines Agency, published in 2012, which states that among the European countries studied the United Kingdom, France, and Italy are the only countries providing financial incentives to develop pediatric drugs [55].

The report of the European Medicines Agency, published in 2016, states that new drugs for the pediatric population are more readily available in Europe and in the United States because these regions have a legal framework regulating the development of drugs for this population. The same document notes that Japan lacks regulations requiring the undertaking of research into new drugs for this population [56].

The forming of global networks, with the aim of reducing the burden of cancer in countries with fewer resources, should be promoted. The more developed nations can help other countries, by sharing their experiences in implementing effective programs for preventing and treating cancer. Some Canadian institutions have contributed to the formation of this network for supporting work on cancer through training health professionals, promoting research into prevention strategies, and supporting means of improving access to diagnosis and treatment [57]. Among networks for supporting work on cancer, the Union for International Cancer Control (UICC) has stood out because of its work in LMIC measuring the global burden resulting from cancer and monitoring and evaluating the data [58, 59].

The WHO has recognized the importance of training researchers and developing infrastructure so that high-quality research may be conducted in LMIC. One example of a successful initiative was in Guatemala, where cancer is the third largest cause of death and the systems for monitoring, preventing, and treating cancer are inadequate. A partnership between the University of Washington, in the United States, and a cancer institute in Guatemala resulted in the training of researchers from Guatemala. Various of these professionals are currently involved in research projects on cancer of the cervix and breast, in consonance with local needs [60].

## 5. Limitations

Data which are incomplete, missing, or inaccurate are limitations related to the databases for registering clinical trials. Although the ICTRP receives data from various registers from a number of countries [16], there are other clinical trial registries, such as those developed by the pharmaceutical industry; as a result, this study may not have captured all cancer clinical trials registered globally. The fact remains, however, that trial registration in WHO-approved registries is broadly endorsed. In addition to this, the data were restricted to the year 2014. For African countries, we defined a period of 10 years for the study, as—in a single year—few clinical trials were registered on the platform. We recognize that these different periods hinder comparison of the data from African countries with those of other countries.

## 6. Conclusions

At a level of significance of 5%, there is a tendency for research into new drugs for treating breast and lung cancer to be undertaken in countries with a lower level of economic development, which may be related to greater exposure to risk factors such as smoking, alcohol, the consumption of industrialized foods, and the adoption of sedentary lifestyles.

The organizations which mostly finance clinical trials for the development of new drugs for treating cancer continue to have little interest in prioritizing resources for financing research into specific types of cancer such as cancers of the cervix, stomach, and liver, which have a major impact on the health of populations of LMIC. It follows that the priorities of international studies in health are not aligned with the public health burden of countries with limited resources.

The participation of pediatric populations in clinical trials has been more common in countries where there are legal frameworks promoting, and financial incentives to encourage, the development of new drugs for this population. Few clinical trials are undertaken in pediatric populations. Although the incidence of cancer in this population is low, research into new drugs is necessary in order to improve these patients' clinical condition.

## Figures and Tables

**Table 1:** Number of clinical trials with patients with cancer by phase of development.

**Country**	**Phase 1**	**Phase 1/2**	**Phase 1/3**	**Phase 2**	**Phase 2/3**	**Phase 3**	**Phase 3/4**	**Phase 4**	**Not Stated**	**Total**
Argentina	1	0	0	7	0	35	0	0	1	44
Brazil	1	2	1	6	0	33	0	2	2	47
Chile	0	0	1	1	0	16	0	1	0	19
China	21	21	0	83	13	63	0	34	19	257
Colombia	0	0	0	3	0	15	0	1	0	19
Denmark	5	5	0	26	1	26	0	4	0	67
Egypt	0	0	0	2	1	10	1	2	0	16
Estonia	0	0	0	2	0	8	0	0	0	10
Finland	1	2	0	8	0	17	0	5	0	33
France	19	14	0	40	3	37	0	3	0	116
Germany	13	8	0	44	5	34	0	3	5	112
India	5	1	1	6	3	27	2	12	20	77
Iran	1	1	0	9	5	6	0	0	7	29
Israel	3	7	0	18	0	43	0	1	0	72
Italy	2	8	1	38	1	34	0	2	1	87
Japan	17	7	0	35	1	13	0	2	12	87
Malaysia	0	0	0	4	1	16	2	1	0	24
Mexico	1	1	1	5	0	38	0	2	0	48
Netherlands	30	25	1	48	1	57	0	13	2	177
Peru	1	0	0	2	0	17	0	0	0	20
Philippines	0	0	1	4	1	25	0	1	0	32
Russia	2	4	1	19	2	51	1	2	0	82
Singapore	8	15	0	19	1	24	0	1	1	69
South Africa	0	0	1	0	0	23	1	0	0	25
South Korea	18	7	1	21	2	27	0	3	7	86
Spain	17	14	1	28	2	36	0	1	0	99
Thailand	2	4	0	11	1	30	2	3	0	53
Turkey	0	1	1	2	0	33	1	0	0	38
Ukraine	2	3	1	3	1	29	0	0	1	40
United K.	24	12	0	32	3	33	0	1	6	111
United States	170	59	1	123	3	35	0	4	32	427

## Data Availability

All data generated or analyzed during this study are included in this published article.
